# Sealing the Leak: Transcatheter Repair of Anterior Mitral Leaflet Perforation With Amplatzer Vascular Plug 4

**DOI:** 10.1016/j.jscai.2025.102634

**Published:** 2025-03-25

**Authors:** Zeryab A. Khan, Cody Carter, Jordan Luli, Nathan Marzlin, Johnathan Hatanelas, Lindsay Castle, Kevin Stiver, Laura Flannery, Steven J. Yakubov, Carlos Sanchez

**Affiliations:** Division of Cardiovascular Medicine, OhioHealth Riverside Methodist Hospital, Columbus, Ohio

**Keywords:** Amplatzer Vascular Plug 4, mitral leaflet perforation, structural, transcatheter-based mitral interventions

## Abstract

We document the first reported use of the Amplatzer Vascular Plug 4 for anterior mitral leaflet perforation repair in a 56-year-old male who had undergone valve-in-valve transcatheter aortic valve replacement. Previously reported cases involving the use of other occluder devices for mitral leaflet perforation repair either required guide catheter or delivery sheath exchanges, arteriovenous wire loop for deployment, or use of mitral transcatheter edge-to-edge repair for leaflet stability. Our case highlights the efficacy of the bilobar-designed Amplatzer Vascular Plug 4 for precise occlusion in anterior mitral leaflet perforation through antegrade-only delivery and safety through the simplification of procedural steps.

## Introduction

The transcatheter-based application of the Amplatzer Vascular Plug (Abbott) in anterior mitral leaflet (AML) perforation provides a potential alternative for high-risk surgical patients. Previous reports of transcatheter-based repair of AML perforation either utilized different occluder devices, including Amplatzer Vascular Plug II, Amplatzer Duct Occluder II, and Amplatzer septal occluder, required guide catheter or delivery sheath exchange, necessitated an arteriovenous wire loop, or mitral transcatheter edge-to-edge repair (MTEER) support.^1^, ^2^, ^3^, ^4^ Our case introduces a novel, solely antegrade technique using 2 Amplatzer Vascular Plug 4 (AVP 4) without MTEER support for repair of leaflet perforation in a patient following recent valve-in-valve transcatheter aortic valve replacement (ViV TAVR).

## Case presentation

**Figure 1:**
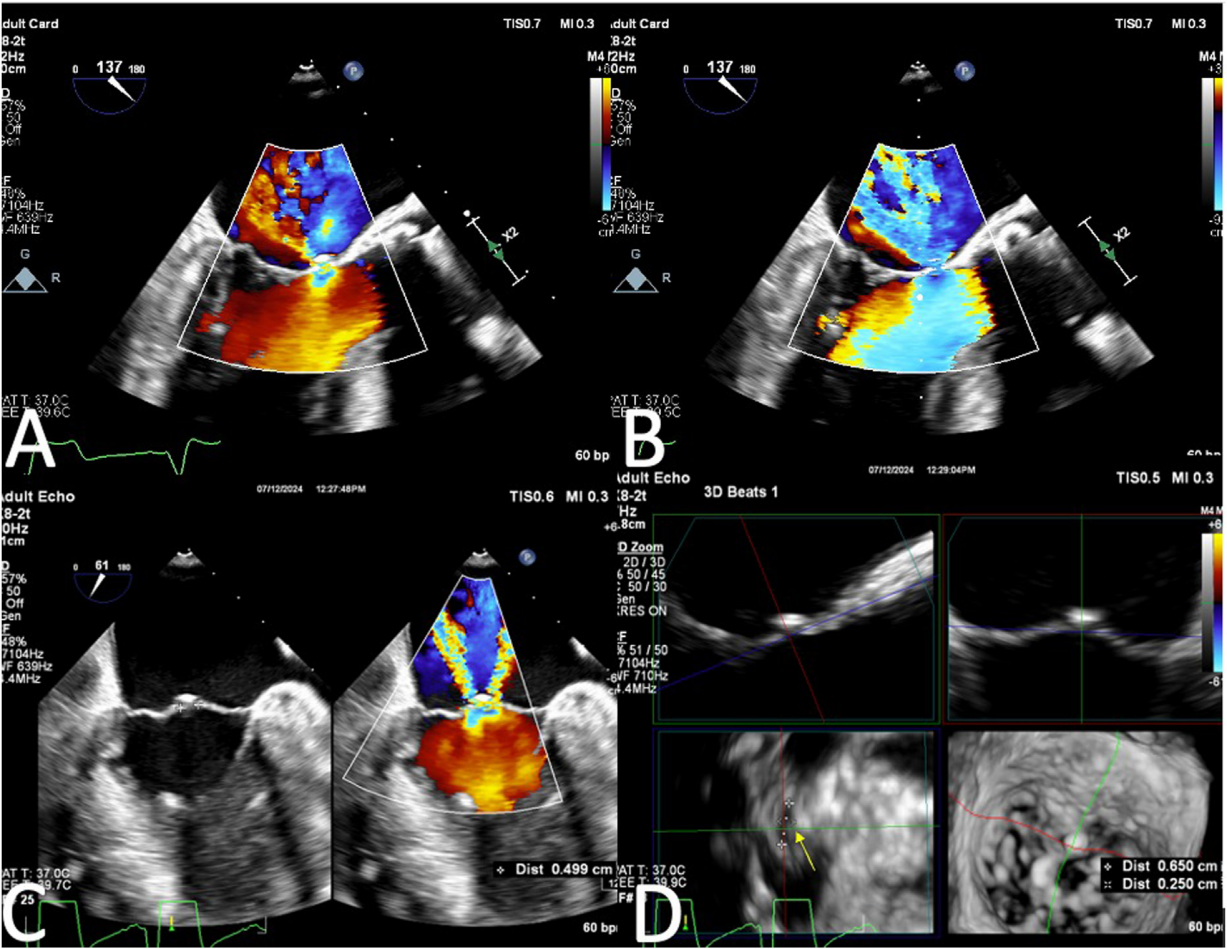
**(A) Transesophageal echocardiogramleft ventricular outflow tractview demonstrating perforation of the basal anterior mitral valve leaflet.** (**B**) PISA radius 1 cm through perforation consistent with severe mitral regurgitation. (**C**) 2D imaging showing the width of the perforation at 0.5 cm. (**D**) Three-dimensional multiplanar reconstruction sizing of the perforation at 0.65 cm × 0.25 cm (yellow arrow).

A 56-year-old man with a history of streptococcal endocarditis requiring surgical aortic valve replacement with a 27 mm Magna bioprosthetic aortic valve (Edwards Lifesciences) 7 years prior presented with progressive dyspnea, orthopnea, and decreased exercise tolerance. Transthoracic echocardiogram (TTE) revealed severe bioprosthetic stenosis with an ejection fraction of 53%, a mean gradient of 46 mm Hg, a peak velocity of 4.5 m/s, and an aortic valve area of 1.0 cm^2^. His symptoms were attributed to bioprosthetic valve failure. Despite undergoing a ViV TAVR (26 mm SAPIEN 3 Ultra valve [Edwards Lifesciences]), which reduced the mean aortic valve gradient to 12 mm Hg, his symptoms persisted. Post-TAVR TTE revealed that his baseline mild mitral regurgitation (MR) had progressed to moderate MR. Further evaluation with transesophageal echocardiogram (TEE) showed moderate to severe MR with a new 0.65 × 0.25 cm perforation in the basal portion of the AML, an effective regurgitant orifice of 0.3 cm^2^, regurgitant volume of 63 mL with systolic flow reversal of the left upper pulmonary vein (Figure 1). It was suspected the perforation was likely an iatrogenic injury from the ViV TAVR procedure. Because the TAV-in-surgical aortic valve was appropriately positioned relative to the aortic annulus, the mechanism of AML perforation was thought to be direct injury from contact between AML and TAV. Another potential mechanism that may have contributed as well was the interaction with the AML and Confida guidewire (Medtronic) during TAV deployment.

**Figure 2:**
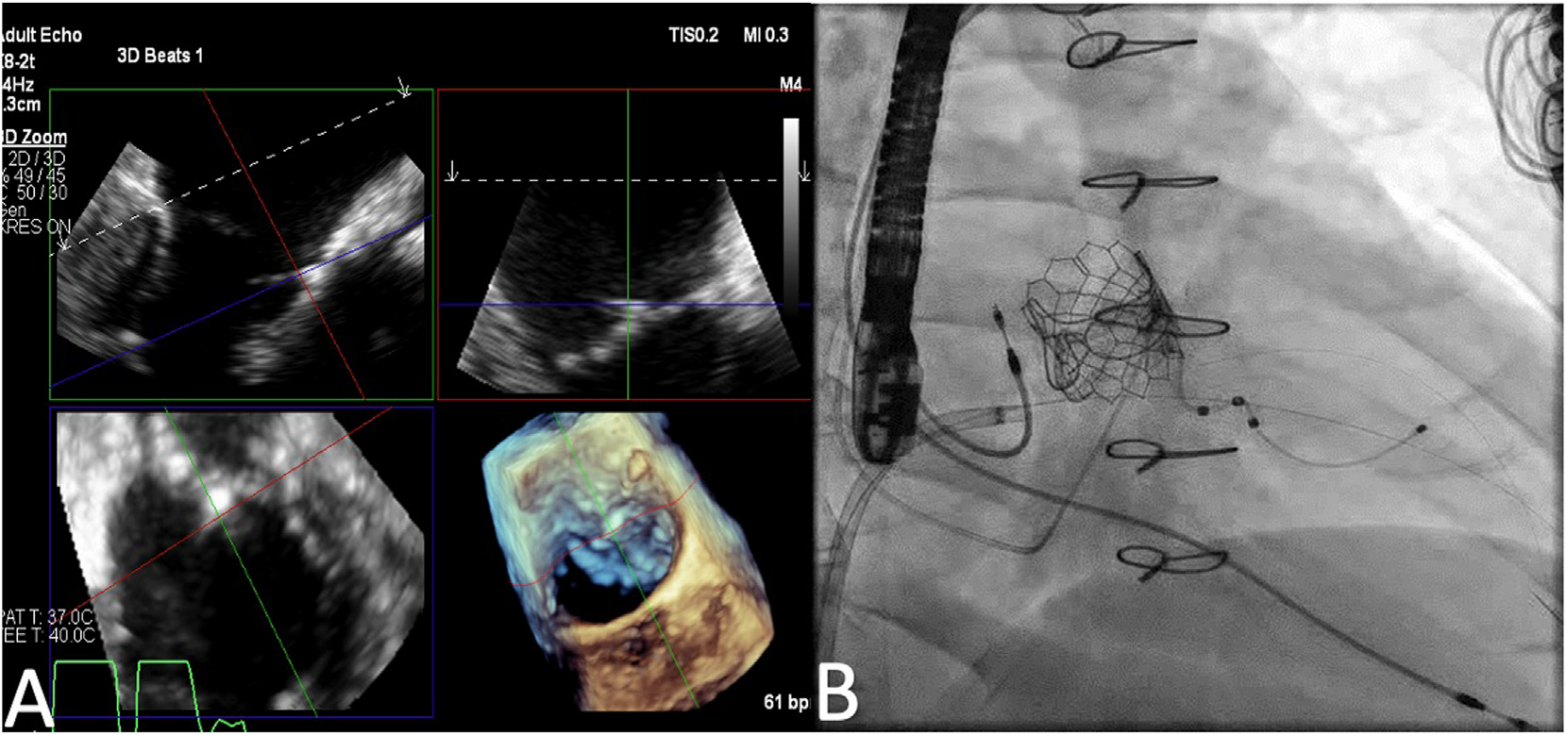
**(A) Real-time 3D multiplanar reconstruction easily guided catheter/wire into perforation.** (**B**) Fluoroscopic view showing transseptal access under real-time 3D multiplanar reconstruction transesophageal echocardiogram guidance, with anterior mitral leaflet perforation cannulated using 2 guidewires.

**Figure 3:**
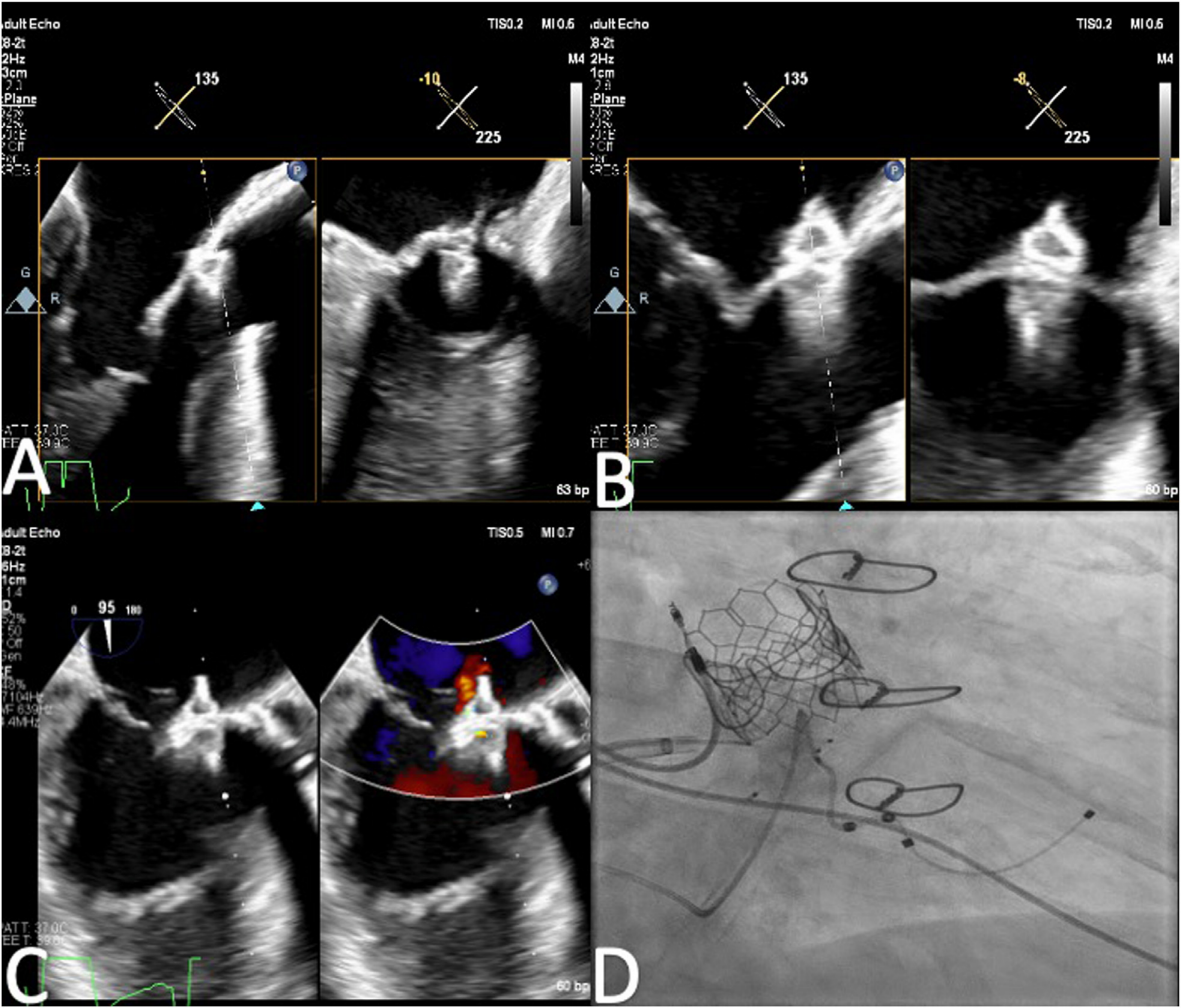
**(A) Transesophageal echocardiogram biplane view of the first Amplatzer Vascular Plug 4 (AVP 4) being deployed.** (**B**) First AVP 4 deployed on the atrial and ventricular sides of the anterior mitral leaflet. (**C**) The second AVP 4 is placed due to moderate residual mitral regurgitation, demonstrating appropriate placement of the second AVP 4 with trace residual mitral regurgitation. (**D**) Fluoroscopic view showing both AVP4.

**Figure 4:**
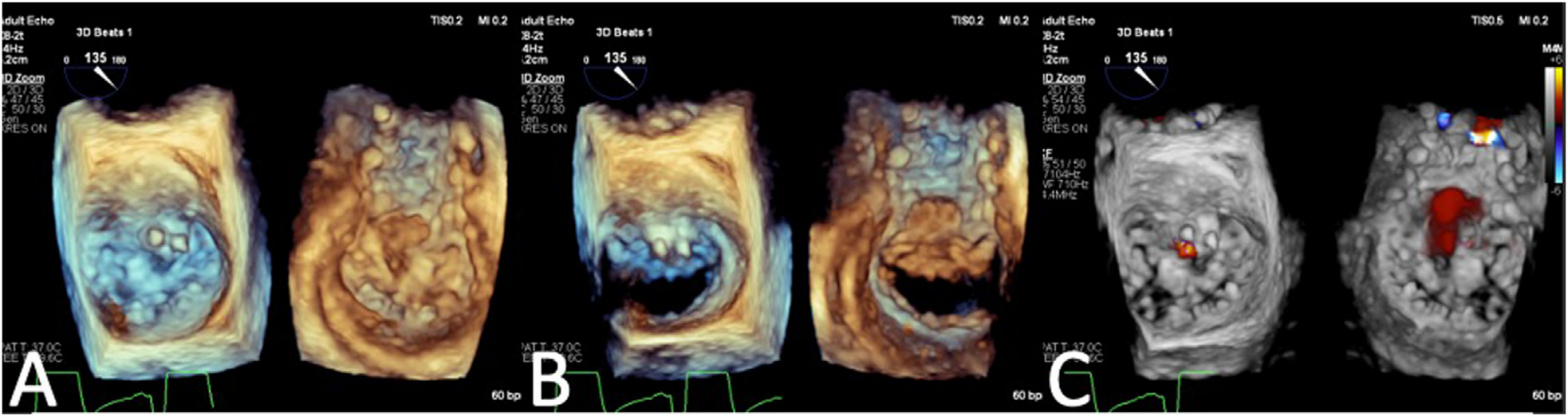
**(A) Three-dimensional transesophageal echocardiogram (TEE) shows both plugs safely positioned on the atrial and ventricular aspects of the mitral leaflets.** (**B**) Three-dimensional TEE shows no impairment of mitral leaflet mobility or mitral valve obstruction during diastole. (**C**) Three-dimensional TEE shows trace residual mitral regurgitation at the end of the case.

Due to the patient’s risk for reoperation, a transcatheter mitral repair was undertaken. Using transseptal access (VersaCross Steerable D1 Curve [Boston Scientific]) under real-time 3D multiplanar reconstruction TEE guidance, the AML perforation was cannulated using 2 guidewires: A 0.014-inch Runthrough and a 0.018-inch Glidewire Gold (both Terumo), with 1 functioning as a safety wire (Figure 2). Based on the manufacturer’s treatable vessel diameter range recommendations for the AVP 4, the 8 mm AVP 4 was selected to be the most appropriate size for the initial device. Delivery of the 8 mm AVP 4 using a 5F multipurpose catheter reduced MR severity from severe to moderate. Subsequently, a 4F multipurpose catheter was advanced over the safety wire to deliver another 7 mm AVP 4. The AML perforation was sealed (Figure 3). A combination of intraprocedural TEE guidance and gentle push-and-pull maneuvers was performed to ensure both lobes of each AVP 4 were securely positioned on each side of the AML to minimize device embolization risk. Intraprocedural TEE confirmed adequate positioning of both AVP 4 with normal mitral leaflet motion, coaptation, trace MR, and mean gradient 1 mm Hg (Figure 4). At 1-month follow-up, the patient was doing well, and TTE confirmed stable positioning of the AVP 4, trace MR, and mitral valve mean gradient of 1 mm Hg.

## Discussion

Our case demonstrates a novel antegrade-only technique using multiple AVP 4 for AML repair. This approach contrasts with prior cases that used other Amplatzer devices which required either guide catheter exchange for delivery of the occluder device, antegrade and retrograde access for arteriovenous wire loop formation for stable deployment, or use of MTEER support for leaflet stability.^5^, ^6^, ^7^ Our technique, guided by multiplanar reconstruction TEE and fluoroscopy, demonstrates the efficient delivery and efficacy of the bilobar-designed AVP 4 for precise occlusion in AML perforation while minimizing procedural complications associated with larger endovascular occluders and dual access.

The AVP 4 distinguishes itself from other devices for leaflet perforation repair with its unique ability to be delivered through 0.014-inch guide wire–compatible diagnostic catheters, eliminating the need for delivery sheath or guide catheter exchanges. The bilobar design allows precise deployment even with unfavorable anatomic leaflet perforations or deflections caused by the MR jet. This, along with the use of a safety wire, allows for a successful antegrade-only approach eliminating the need for the creation of an arteriovenous loop for sheath delivery for device deployment. Furthermore, delivering the AVP 4 through a smaller 4 to 5 F catheter eliminates the need for MTEER stabilization in order to reduce leaflet tension and prevent potential worsening of the leaflet perforation.^8^

Our use of multiple AVP 4 in an antegrade-only technique highlights the potential to effectively address complex iatrogenic valvular injuries following surgical or structural interventions in a percutaneous manner.^9^ This may be especially useful in leaflet injuries following valvular endocarditis. Our device selection and technique is a demonstration of simplifying the closure of leaflet perforation.

## Conclusion

This case demonstrates an alternative, simplified technique for AVP repair in mitral leaflet perforation. The exclusive antegrade approach with AVP 4 using a small diagnostic catheter for device delivery with coronary artery wire cannulation of AML perforation advances the safety of this procedure. Long-term follow-up for the durability of this approach is essential.
